# Pyorrhea Alveolaris as a Cause of Colitis

**Published:** 1910-06-15

**Authors:** 


### PYORRHEA ALVEOLARIS AS A CAUSE OF COLITIS.

In the course of an article on "Seven Cases of Appendicostomy for Various Forms of Colitis," published in the *British Medical Journal*, of Oct. 30th, Mr. Frederick C.

Wallis, F. R. C. S., Surgeon to Charring Cross Hospital, relates seven cases of appendicostomy which he regards as a typical class of case in which the operation is of most benefit.

The following (Case 2) is of special interest from a dental point of view:

T., aged 45, was brought to me by Dr. Gregory, of Redcliffe Gardens, in July, 1908. The history was that she had been passing blood and mucous since Christmas. There was slight abdominal pain. She was losing flesh and had become anemic. On examining the mouth there was a marked condition of pyorrhea alveolaris.

A sigmoidoscopic examination was made under an anesthetic on July 2nd, 1908, and it was found that the upper rectum and the sigmoid as far as the sigmoidoscope would go were ulcerated in patches and generally congested.

The *Streptococcus longus* was cultivated from a swabbing taken from the discharge in the sigmoidoscope. This strongly suggested that the pyorrhea alveolaris was the cause of the colitis. The condition of the gums was immediately treated, and the patient was dieted and put on lacto-bacilline.

*Operation.*—Appendicostomy was performed on July 10th. The colon was washed through with several pints of sodium bicarbonate one dram to the pint, on the operation table, and for the succeeding seven days four pints of this solution were washed through twice a day. For the last five days there was no blood nor mucous in the washout.

The patient went into the country shortly afterwards, and the appendicostomy opening was allowed to close up. She has remained perfectly well ever since.—*Dental Surgeon*, London.

